# The effect of tert-butylhydroquinone on anxiolytic- and antidepressant-like behaviors induced by post-traumatic stress disorder: A behavioral and molecular study

**DOI:** 10.22038/ijbms.2025.87679.18939

**Published:** 2026

**Authors:** Samaneh Nabavi, Mohammad-Reza Zarrindast, Fariba Khodagholi, Mohammad Nasehi, Solmaz Khalifeh

**Affiliations:** 1 Institute for Cognitive Science Studies, Tehran, Iran; 2 Department of Pharmacology, School of Medicine, Tehran University of Medical Sciences, Tehran, Iran; 3 Neuroscience Research Center, Shahid Beheshti University of Medical Sciences, Tehran, Iran; 4 Cognitive and Neuroscience Research Center, Tehran Medical Sciences, Islamic Azad University, Iran; 5 Department of Physiology, Tehran Medical Sciences, Islamic Azad University, Tehran, Iran

**Keywords:** Anxiety, Depression, Neuroinflammation, Post-traumatic stress - disorder (PTSD), Tert-butylhydroquinone (tBHQ)

## Abstract

**Objective(s):**

Post-traumatic stress disorder (PTSD) is a crippling mental illness that commonly co-occurs with anxiety and depression. Recent studies have established a link between neuroinflammation and the development of PTSD. Studies have revealed that tert-butylhydroquinone (tBHQ) exhibits anti-inflammatory effects. This research investigated the impact of tBHQ on the amelioration of PTSD-induced depression- and anxiety-like behaviors with respect to amygdala and hippocampal MAO-A, MAO-B, IL-6, IL-10, and glucocorticoid receptor in rats.

**Materials and Methods:**

PTSD was triggered through the use of Single Prolonged Stress (SPS). The Elevated Plus Maze (EPM) and Forced Swim Test (FST) were employed to evaluate anxiety and depression, respectively. Protein assessment utilized Western blot assay for IL-6, IL-10, and the glucocorticoid receptor, in addition to enzyme-linked immunosorbent assay (ELISA) for MAO-A and MAO-B.

**Results:**

The data demonstrated that treatment with tBHQ ameliorates the depression- and anxiety-like behaviors in rats with PTSD. The ELISA findings indicated a rise in both MAO-A and B proteins in the hippocampus and amygdala due to PTSD, which was counteracted by a subthreshold amount of tBHQ. Additionally, the Western blot assay techniques revealed that PTSD led to an elevation in IL-6 levels, a pro-inflammatory cytokine, and a reduction in IL-10 levels, along with a decrease in glucocorticoid receptor expression in both brain regions, which was also counteracted by a subthreshold amount of tBHQ.

**Conclusion:**

These findings suggested that the beneficial effect of tBHQ on anxiety and depression induced by PTSD through hippocampal and amygdala MAO-A, MAO-B, IL-6, and IL-10, and glucocorticoid receptor.

## Introduction

With a lifetime prevalence of 5.6% worldwide, post-traumatic stress disorder (PTSD) is a profoundly incapacitating mental health condition. It is typically characterized by a cluster of symptoms that manifest after exposure to events that pose a significant risk to one’s life, such as violence, conflict, serious injury, or death, including invasion or re-experience, avoidance, unfavorable changes in mood, cognition, and excessive stress (1, 2). PTSD has been linked to the emergence of chronic illnesses and other detrimental physical health issues, including autoimmune disease and cardiovascular disease (3, 4). Additionally, patients with PTSD exhibit a greater incidence of comorbidities, including depression, anxiety, and several other psychiatric disorders (5). It has been established that neuroendocrine, psychophysiological, and neurobiological abnormalities are associated with the development and maintenance of PTSD. Furthermore, it has been associated with a number of physiological complications, including oxidative stress, neuroinflammation, hypothalamic pituitary adrenal (HPA) dysfunction, and cellular senescence. In PTSD, the HPA axis is described in detail as being dysregulated (6-8). Patients with PTSD have decreased sensitivity to glucocorticoid receptors (GRs), which may lead to inflammation. An augmented susceptibility to PTSD is linked to diminished concentrations of glucocorticoids, which are synthesized by the adrenal glands (8-11).

The interconnections in structure and function between the amygdala and various brain regions associated with emotions, including the prefrontal cortex, hippocampus, and anterior cingulate cortex, are vital for understanding anxiety and depressive behaviors triggered by stress (12-14). Research has repeatedly demonstrated that the strengthened prefrontal cortex-amygdala pathway plays a significant role in psychiatric disorders associated with inflammation. For instance, elevated amygdala activity correlates with heightened inflammation in response to social stressors. People demonstrating a robust connection between the amygdala and prefrontal cortex show increased inflammation in response to stressors (15).

Neuroinflammation is characterized by the recruitment of monocytes into the brain, the activation of local glial cells, and the release of pro-inflammatory cytokines in response to infection and damage (8, 16). The adverse impact of prolonged, heightened inflammatory reactions in the central nervous system is expected to be observed in glial cells, neurons, and signaling molecules. Hence, it has been postulated that a reciprocal causal association exists between inflammation and PTSD (8, 17). Understanding the relationship between PTSD and neuroinflammation is critical because it can provide insights into the potential involvement of neuroinflammation in the vulnerability to PTSD, as well as the link between PTSD and various enduring conditions. Moreover, neuroinflammation can impact treatment or preventative strategies. Neuroinflammation can be exacerbated by stress-induced HPA dysregulation (8), and neuroinflammation inhibition can reduce anxiety-like behaviors (18, 19). PTSD patients have higher levels of pro-inflammatory cytokines in their blood and brains than healthy individuals, according to multiple studies. On the other hand, a number of research groups have conducted comparisons regarding the concentrations of cytokines that inhibit inflammation, such as interleukin 10 (IL-10) and interleukin 4 (IL-4) (20). Anti-inflammatory medications have been demonstrated to lessen depression-like behaviors, and neuroinflammation may potentially play a significant role in depression etiology (21).

Monoamine oxidase A (MAO-A) and monoamine oxidase B (MAO-B) may have a significant impact on PTSD. More positive, psychotic, and depressive symptoms are correlated with reduced platelet MAO-B activity in PTSD. Conversely, an increase in noradrenergic signaling may be facilitated by MAO-A gene hypermethylation. Additionally, MAO intron 13 and MAO-B polymorphisms may be linked to PTSD symptoms. MAO-B and MAO intron 13 polymorphisms may also be associated with PTSD symptoms (22-24)

**Figure 1:**
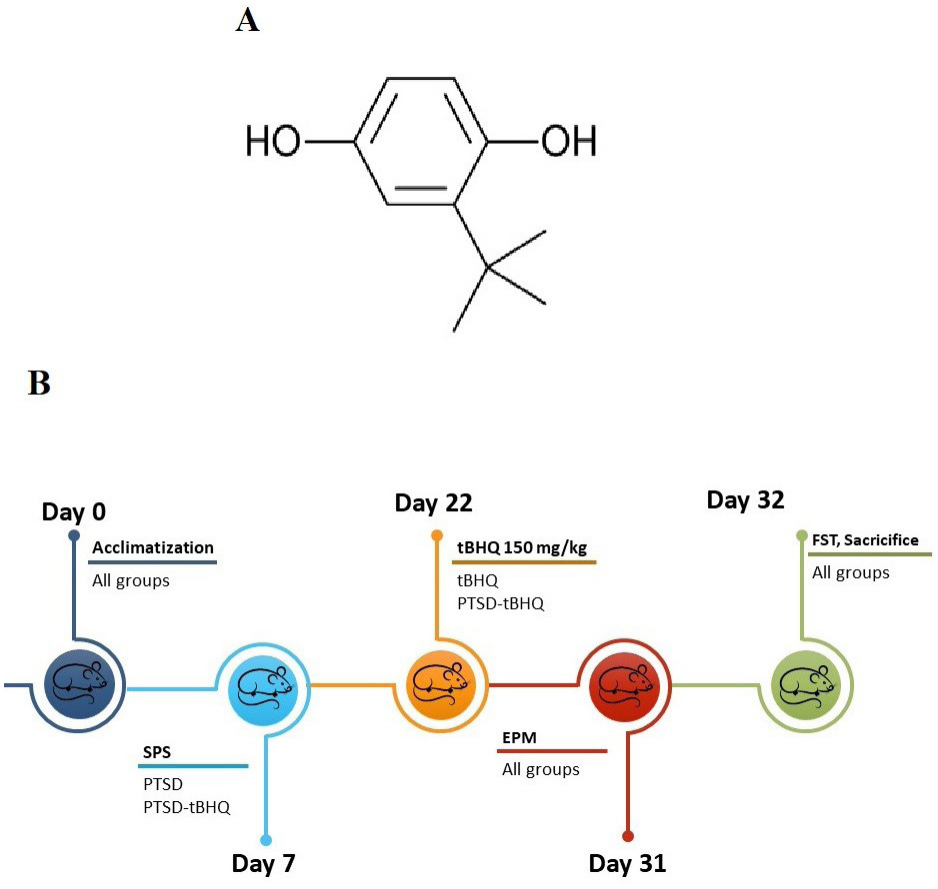
A. The chemical structure of tBHQ and B. Experimental design and timeline

The synthetic anti-oxidant tert-butylhydroquinone (tBHQ) is well-known for being inexpensive, highly effective, and requiring a low dosage. tBHQ can protect animal cells and tissues from oxidative stress and inflammation-induced dysfunction (21), in addition to its safety and outstanding anti-oxidant activity (Figure 1A). 

The strong association between inflammation and changes in Nrf2-dependent redox homeostasis has been widely recognized. The administration of tBHQ elevated GPx and SOD levels while concurrently reducing NF-κB activity. Delays in the synthesis of oxidative products and inflammatory cytokines have provided evidence that these effects confer protection against oxidative damage. Furthermore, the anti-oxidant mechanism was restored by tBHQ treatment. Previous studies have demonstrated that tBHQ effectively mitigates inflammation induced by fine particulate matter by facilitating Nrf2 transcriptional activity (21). There is currently no scientifically validated treatment for PTSD. Moreover, the underlying mechanism is not wholly transparent. As a result, it is essential to demonstrate the pathological process underlying PTSD and to continue researching more comprehensive methods for its prevention and treatment.

By utilizing the Elevated Plus Maze (EPM) and Forced Swim Test (FST), we assessed the potential therapeutic applications of tBHQ in rats with PTSD. Due to the influence of IL-6, IL-10, GR, MAO-A, and MAO-B in PTSD, we also evaluated their expression in the hippocampus and amygdala, two critical brain regions implicated in stress and stress-related behavior (25, 26).

## Materials and Methods

### Animals

For the experiments, 40 adult male Wistar rats (180-200 g) were utilized. They were kept in groups of four in cages. The rats were purchased from the Pasteur Institute of Iran. The animals were provided with constant access to food and water. They were kept in standard conditions (23 degrees Celsius, 50% humidity, and no disturbance) with a 12-hr light/dark cycle (lights on at 7:00 AM). The Research and Ethics Committee of the Institute for Cognitive Science Studies (ICSS) has approved the experimental methods described in this publication (NIH publication number 80-23).

### Experimental design

The experimental procedure is illustrated in Figure 1B. There were four groups of rats (n = 10 per group) based on randomization: the control group, the PTSD group, the tBHQ treatment group, and the PTSD + tBHQ treatment group. The anxiolytic and antidepressant effects of tBHQ were assessed using the EPM and FST. The expression levels of GR, IL-10, and IL-6 were evaluated by western blotting (n = 3 per group). ELISA was used to determine corticosterone concentration in serum, MAO-A and MAO-B levels in the amygdala and hippocampus (n = 3 per group).

### Drug treatment

Oral gavage was used to administer 150 mg/kg of tBHQ (Sigma, USA) in corn oil to animals daily for eight days (27).

### Single prolonged stress (SPS)

In studies on PTSD, SPS is a popular animal model (28, 29). Following the acclimatization period, each rat was placed in a tail-gate restrainer for two hours. To achieve complete immobilization, the size was adjusted to the individual’s size. Following restraint, each rat was forced to swim for 20 min in a transparent acrylic cylinder (20 cm in diameter, 45 cm in height, with approximately 25 cm of 23–24 °C water). Each individual was exposed to diethyl ether for 15 min, or until they lost consciousness. The control group was handled and housed alongside the SPS rats.

### Forced swim test (FST)

A forced swimming test was conducted in a 20 cm diameter, transparent, cylindrical plastic tank. The tank was 45 cm tall, and tap water had been poured into it to a depth of 30 cm (24 ± 1 °C). Rats were gently lowered into the water the day before the test for more accurate findings. After 15 min, they were removed, allowed to dry, and then returned to their cages. Rats were placed in the cylindrical tank once more for the 6-min test. Total immobility time was calculated as the time the rat floated motionless, making only slight movements to keep its head above water (30).

### Elevated plus maze (EPM)

The EPM was utilized to evaluate the anxiety-like behavior of the rats. Rats were allowed to explore their surroundings in the EPM test setup during darkness and quiet. The device was cross-shaped and composed of dark Plexiglas. It had a central point, two open arms positioned opposite one another, and two enclosed arms of equal dimensions. The maze was lighted by a modest light source and stood 50 centimeters above the ground. The number of entrances into the open and closed arms was counted during the five minutes of observation, and the amount of time that each rat spent on the open arm was calculated. Arm entrance is defined as the entry of all four limbs into an arm. Between each test, the equipment was cleaned with 70% ethanol. The anxiety index was computed utilizing the subsequent equation, which incorporates the behavioral measures of the EPM (31):

$1-[\frac{\left(\frac{\mathrm{Time spent inm the open arms}}{\mathrm{Total time on the maze}}+\frac{\mathrm{Number of enteries to the open arms}}{\mathrm{Total exploration on the maze}}\right)}{2}]$

### Brain dissection

Following the completion of the behavioral assessments, the rats were sacrificed through decapitation. Subsequent to the decapitation, the amygdala and hippocampus were meticulously excised from the surrounding brain tissue by sectioning the midline of the brain, thereby facilitating the division of the cerebral hemispheres. The isolated amygdala and hippocampus underwent a thorough wash with saline solution. Subsequently, the specimens were transferred into an RNase-free microtube. The samples were then promptly stored in a freezer at -70 °C until required for subsequent analysis.

### Western blot assay

The hippocampus and amygdala tissues (three samples per group) were homogenized in the presence of lysis buffer to extract total protein. The supernatant was collected after centrifugation (13,000 g, 4 °C, 30 min), and the protein concentration was determined using the Bradford assay. Equal amounts of protein (60 μg) were loaded onto a 12.5% SDS-PAGE gel, separated by electrophoresis, and transferred to PVDF membranes. To block non-specific binding, membranes were incubated with 2% nonfat dry milk in blocking solution for 75 min at room temperature. Subsequently, the membranes were incubated overnight at 4 °C with primary antibodies against IL-6, IL-10, and GR. After washing with Tris-buffered saline containing Tween-20, the membranes were incubated with horseradish peroxidase-conjugated secondary antibodies (90 min, room temperature). Protein bands were visualized using the conventional autoradiography method. PVDF membranes were incubated with ECL detection reagents (Parstous, Iran), exposed to X-ray film (FujiFilm, Japan) in a cassette, and developed and fixed in a darkroom using photographic developer and fixer solutions (Tetenal, Germany). Protein band densities were quantified using ImageJ software (NIH, USA).

### Enzyme-linked immunosorbent assay (ELISA)

The ELISA was employed to quantify the concentrations of MAO-A and MAO-B in the amygdala and hippocampus (three samples per group), and corticosterone in serum. The measurements and procedures were carried out in accordance with the manufacturer’s instructions for the corticosterone, MAO-A, and MAO-B ELISA kits. ELISA absorbance was measured using a microplate reader (DANA-3200, Garni Medical Engineering Co., Iran).

### Statistical analysis

The normality of data for each group was assessed by the Kolmogorov-Smirnov test, and using the mean and standard deviation (SD), the results were presented. The graphs were analyzed and created using GraphPad Prism 9.0 (GraphPad, CA, United States). Two-way analysis of variance (two-way ANOVA) was utilized for statistical comparisons. To compare means, the Tukey test for multiple comparisons was used. *P*<0.05 served as the statistical significance threshold.

## Results

### tBHQ effects on anxiety-like behavior

**Figure 2:**
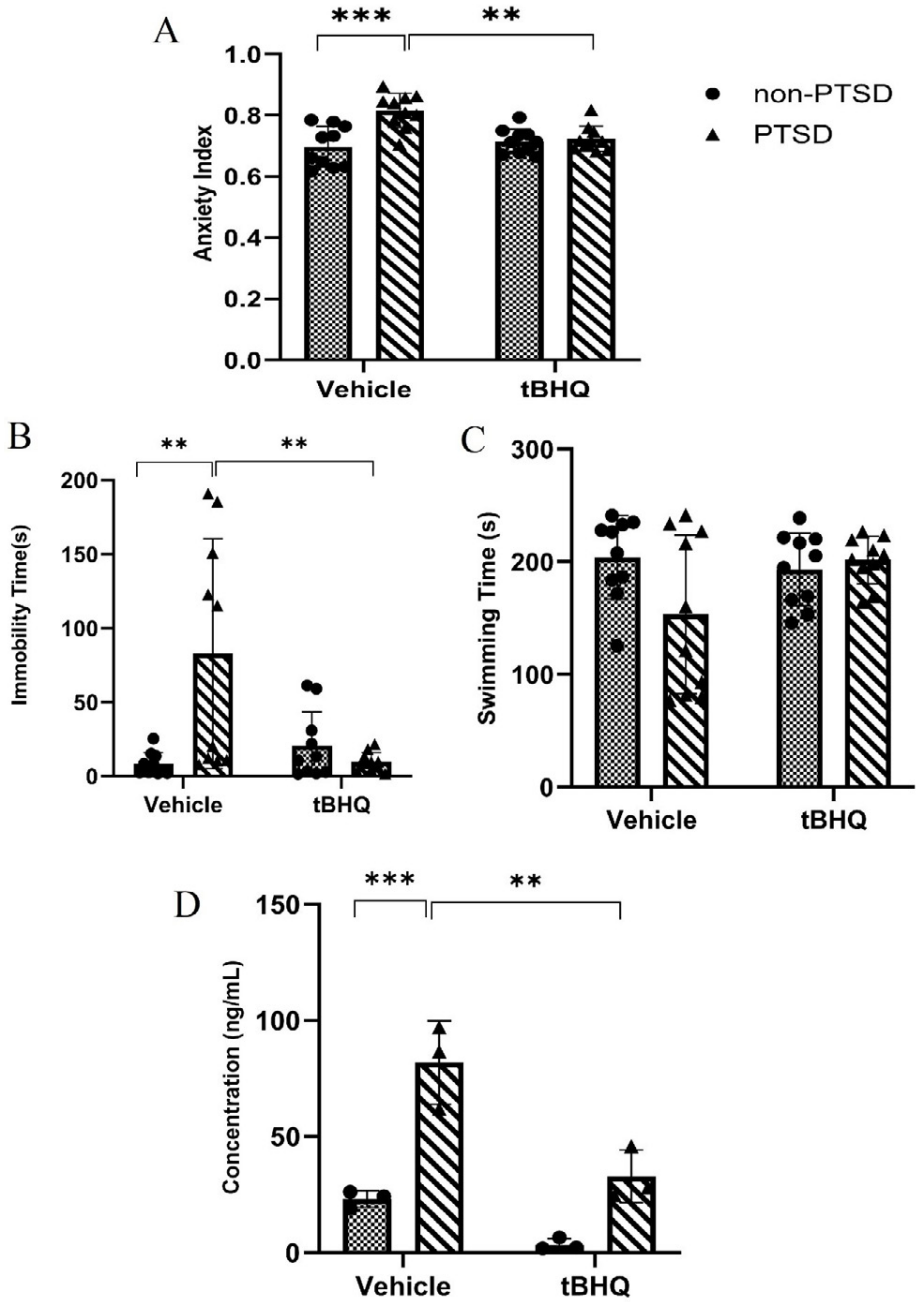
Effects of tBHQ on rat Anxiety Index, immobility time, swimming time and corticosterone

The results of the anxiety index were illustrated in Figure 2A. The main effects of PTSD were found [*F (1,36)* = 5.19;* P*<0.05] and tBHQ [*F* (1,36) = 14.95;* P*<0.001], in addition to their interaction [*F (1,36)* = 14.95; *P*<0.001] and [*F (1,36)* = 10.83; *P*<0.01]. It was discovered that tBHQ treatment significantly decreased the anxiety index in rats with PTSD (*P*<0.01). 

### tBHQ effects on depression-like behavior

Figure 2B illustrates the time spent immobilized in four groups. It demonstrated the main effects of PTSD [*F* (1,36) = 5.541; *P*<0.05] and tBHQ [*F* (1,36) = 6.040; *P*<0.05] in addition to their interaction [*F* (1,36) = 10.93; *P<0.01*]. Treatment with tBHQ significantly reduced immobility time in rats with PTSD (*P*<0.01). The swimming duration is depicted in Figure 2C. It discovered the main effects of PTSD [*F* (1,36) = 1.799; *P*>0.05] and tBHQ [*F* (1,36) = 2.279; *P*>0.05], as well as their interaction [*F* (1,36) = 4.442; *P<0.05*]. The administration of tBHQ did not affect the swimming time in rats with PTSD (*P*>0.05).

### tBHQ effects on the concentration of corticosterone in serum

Figure 2D illustrates the corticosterone concentration in serum. It showed the interaction between PTSD and tBHQ [*F* (1,8) = 5.425; *P*<0.05], in addition to their respective main effects [*F* (1,8) = 30.03;* P*<0.001] and [*F* (1,8) = 49.23; *P*<0.001]. The treatment with tBHQ was found to significantly reduce corticosterone levels in the serum of rats with PTSD.

### tBHQ effects on the expression level of MAOs (MAO-A and MAO-B) in the amygdala

**Figure 3:**
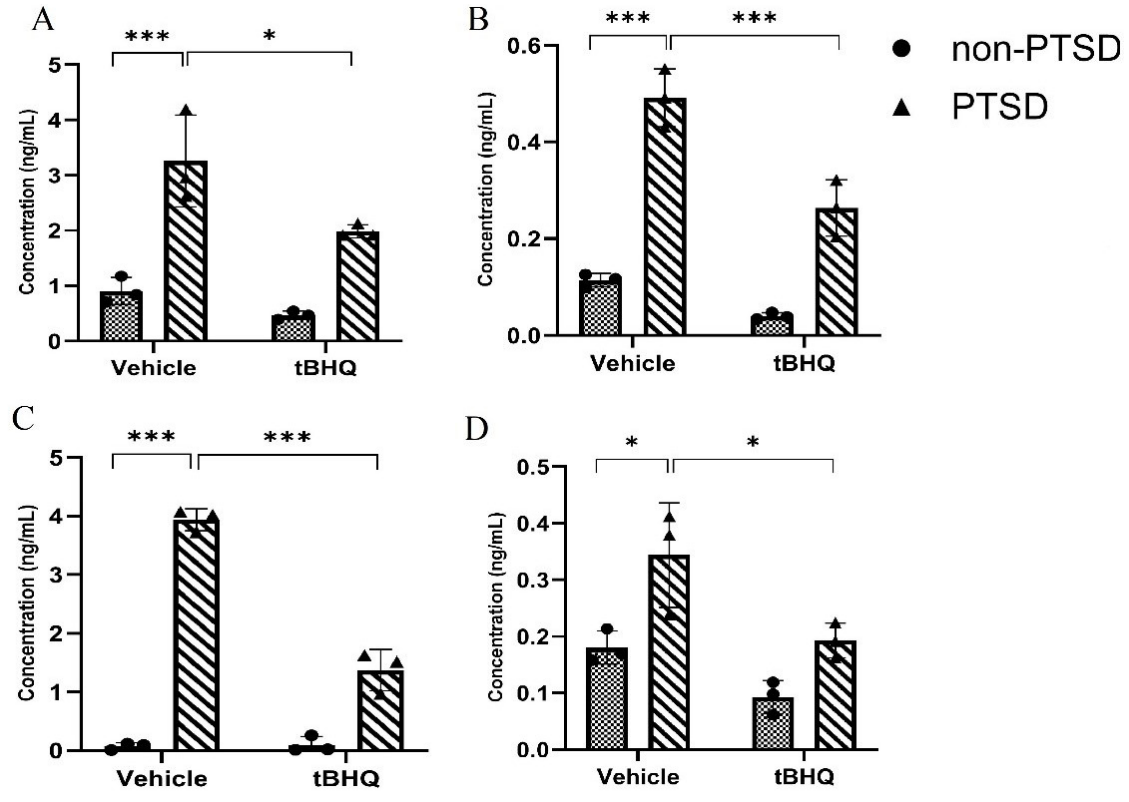
Effects of tBHQ on the concentration of MAOs in rats

PTSD increased the expression of MAO-A and MAO-B, as shown in Figure 3a, b [*F* (1,8) = 11.45; *P*<0.01] and [*F* (1,8) = 37.72; *P*<0.01], respectively. The concentrations of MAO-A and MAO-B decreased in response to tBHQ, with respective values of [*F* (1,8) = 58.47; *P*<0.001] and [*F* (1,8) = 149.4; *P*<0.001]. tBHQ significantly decreased the expression of MAO-A and MAO-B in rats with PTSD, as indicated by the interaction between these variables [*F* (1,8) = 2.709; *P*>0.05 and [*F* (1,8) = 9.787; *P<*0.05], respectively.

### tBHQ effects on the expression level of MAOs (MAO-A and MAO-B) in the hippocampus

As shown in Figure 3c, d, PTSD increased the concentrations of MAO-A and MAO-B, [*F* (1,8) = 106.6; *P<0.0*01] and [*F* (1,8) = 15.25; *P*<0.01], respectively. MAO-A and MAO-B concentrations were reduced by tBHQ, [*F* (1,8) = 435.8; *P*<0.001] and [*F* (1,8) = 18.63; *P<0.01*], respectively. Treatment with tBHQ significantly decreased MAO-A and MAO-B concentration in rats with PTSD, [*F* (1,8) = 111.1; *P*<0.001] and [*F* (1,8) = 1.084;* P*>0.05], respectively.

### tBHQ effects on the expression level of GR in the amygdala

**Figure 4:**
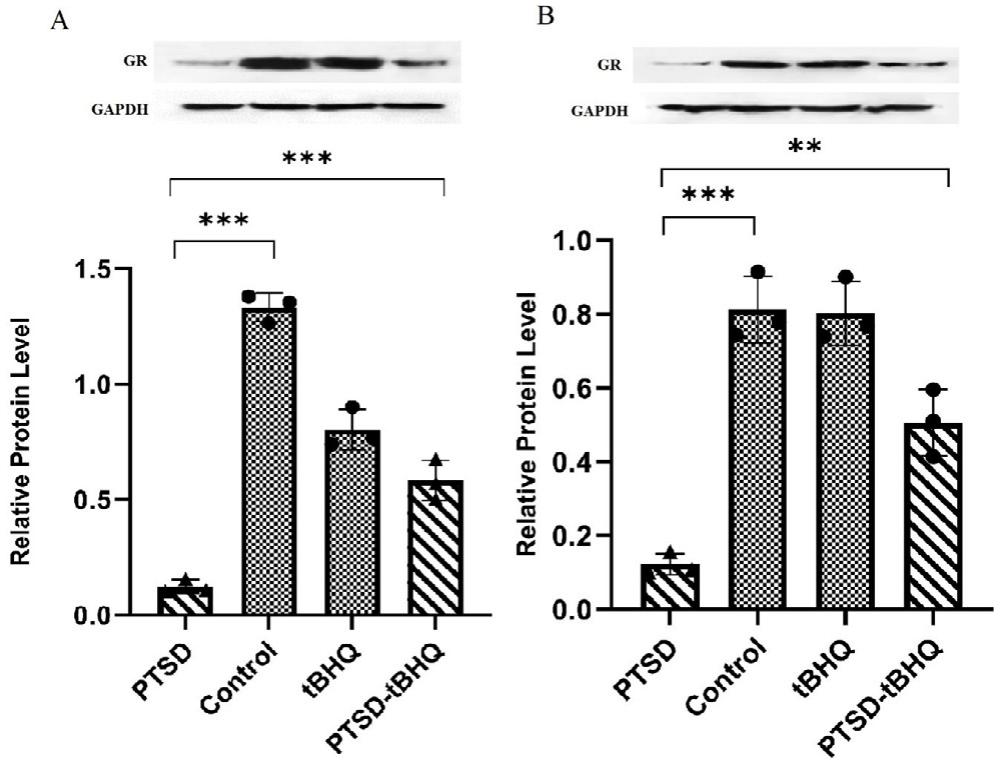
Effects of tBHQ on rat GR expression

As shown in Figure 4a, PTSD induced a decrease in GR expression [*F* (1,8) = 0.7351; *P>0.05*], whereas tBHQ induced an increase in GR expression [*F* (1,8) = 311.8; *P<*0.001]. The results of the interaction between these two factors revealed that the treatment with tBHQ markedly increased GR expression [*F* (1,8) = 149.7; *P*<0.001].

### tBHQ effects on the expression level of GR in the hippocampus

As shown in Figure 4b, PTSD induced a decrease [*F* (1,8) = 16.94; *P*<0.01] and tBHQ induced an increase in GR expression [*F* (1,8) = 117.4; *P*<0.001]. The results of the interaction between these two factors revealed that the treatment with tBHQ markedly increased GR expression [*F* (1,8) = 18.72; *P*<0.01] in rats with PTSD.

### tBHQ effects on the expression level of IL-6 and IL-10 in the amygdala

**Figure 5:**
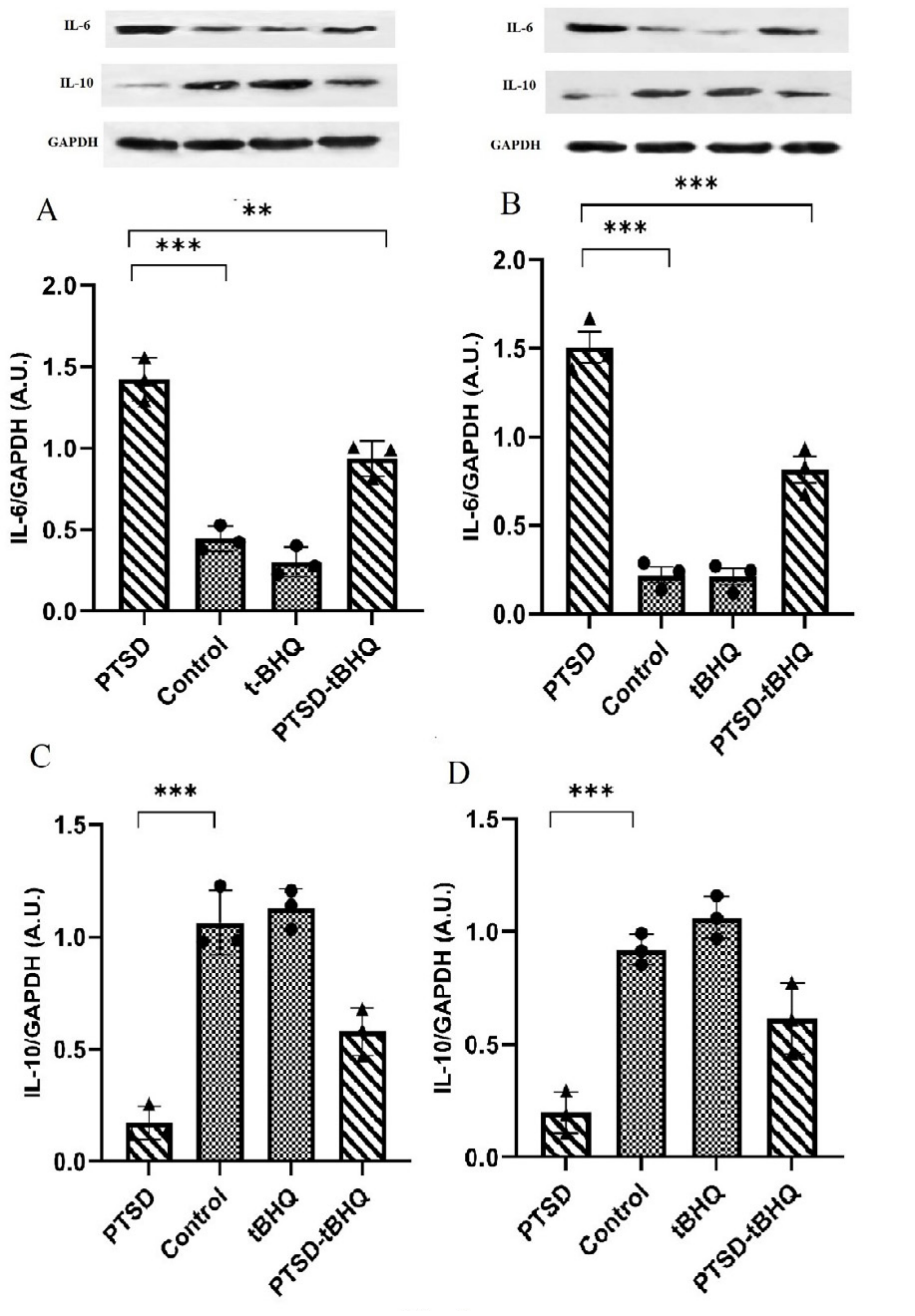
Effects of tBHQ on IL-6 and IL-10 expression in rats

The expression level of IL-6 was illustrated in Figure 5a. The findings showed that PTSD and tBHQ had significant main effects [*F* (1,8) = 27.22; *P*<0.001] and [*F* (1,8) = 178.9; *P*<0.05], respectively, in addition to their interaction [*F* (1,8) = 8.186; *P*<0.05]. tBHQ decreased the expression of IL-6 that was induced by PTSD (*P*<0.01), according to the findings. Figure 5c illustrates the expression level of IL-10. The results indicated that IL-10 expression played a substantial role in both PTSD and tBHQ levels [*F* (1,8) = 14.90; *P*<0.01] and [*F* (1,8) = 139.9; *P*<0.001]. Additionally, IL-10 expression influenced the interaction between these variables [*F* (1,8) = 7.944; *P*<0.05]. The findings revealed that the administration of tBHQ treatment mitigated the reduction in IL-10 expression caused by PTSD (*P*<0.01). 

### tBHQ effects on the expression level of IL-6 and IL-10 in the hippocampus

Figure 5b depicts the expression level of IL-6. The findings demonstrated that PTSD [*F* (1,8) = 28.63; *P*<0.001] and tBHQ [*F* (1,8) = 208.3; *P*<0.001] had significant impacts, as did their interaction [*F* (1,8) = 26.96; *P*<0.001]. The results showed that tBHQ reduced the IL-6 expression caused by PTSD (*P*<0.001). Figure 5d depicts the expression level of IL-10. The findings demonstrated that PTSD [*F* (1,8) = 20.14; *P*<0.01] and tBHQ [*F* (1,8) = 88.28; *P*<0.001], as did their interaction [*F* (1,8) = 4.872; *P*>0.05]. The results demonstrated that tBHQ treatment compensated for the PTSD-induced decrease in IL-10 expression (*P*<0.01).

## Discussion

Anxiety and depressive-like behavior are the primary signs and symptoms of PTSD (32). A growing body of evidence from blood biomarkers, genetic associations, and studies on DNA methylation indicates that inflammatory mechanisms are fundamental to the pathogenesis of PTSD. Numerous fundamental and clinical studies have investigated the mechanisms by which elevated inflammation leads to the onset of PTSD (33, 34). A number of anti-inflammatory medications have also been investigated for their therapeutic potential in PTSD (35, 36). Cumulative evidence from the extant body of literature supports the anti-inflammatory properties of tBHQ (21). The therapeutic potential of tBHQ in mitigating depression induced by lipopolysaccharide (LPS) was examined in mice in a recent study (37).

To the best of our knowledge, this is the initial investigation into the efficacy of tBHQ as an anxiety-reducing agent and antidepressant in an animal model of PTSD. We utilized behavioral and molecular evidence to investigate these effects. Our behavioral and molecular data demonstrated that tBHQ is a viable treatment option for depression and anxiety behaviors induced by PTSD in rats. 

We adopted the “anxiety index” as a suitable metric for measuring anxiety based on a number of studies conducted in this field. Its values range from 0 to 1, and as the index rises, so does anxious behavior (31). We found that rats with PTSD are more anxious than healthy rats, which is consistent with previous research on the behaviors induced by PTSD-induced anxiety, and tBHQ reduced the elevated anxiety index in rats with PTSD. 

Immobility and swimming time are two practical criteria for researching depression-like behaviors caused by PTSD (38). Similarly, we found that rats with PTSD exhibit depression-like behavior through an increase in immobility, despite the fact that swimming time was unaffected. Numerous studies have shown that corticosterone levels rise during PTSD (32). The compulsory maintenance of elevated cortisol levels is likely to contribute to anxiety-like behavior. A comparable mechanism could potentially be accountable for the advancement or intensification of PTSD in human individuals (28). In spite of a notable elevation in serum corticosterone levels in rats with PTSD, the administration of tBHQ leads to a significant reduction in corticosterone concentration. This positive effect of tBHQ for the treatment of anxiety-like behavior in the animal model of PTSD in rats can be confirmed by the significant association between the anxiety index and the molecular data. It appears that tBHQ normalizes behavioral and neurochemical responses by lowering blood corticosterone levels, thereby preventing HPA axis-related psychological dysfunction. 

Extensive research has been conducted on depressive symptoms induced by PTSD utilizing the MAO assessment (22). MAO-A and MAO-B concentrations in the hippocampus and amygdala were determined. The administration of tBHQ decreased their elevated concentrations due to PTSD. These results indicate that tBHQ can alleviate depressive-like behavior.

The GRs play essential roles in regulating the negative feedback loop of the HPA axis and are widely distributed throughout the hippocampus and amygdala. Consistently elevated levels of glucocorticoids (corticosterone in rodents) contribute to the malfunction of the HPA axis by causing desensitization and down-regulation of GRs (39). The term “glucocorticoid resistance” refers to the reduced glucocorticoid response that has been repeatedly observed in depressive disorders and PTSD-related behaviors (40). Multiple studies indicate that antidepressants tend to enhance GR function in humans, animals, and cells. The administration of tBHQ reversed a significant decrease in GR levels in the amygdala and hippocampus of rats with PTSD, suggesting that this medication may be capable of restoring the negative feedback control of the HPA axis. The findings presented here align with the conclusions drawn from a multitude of studies that antidepressants induce the upregulation of GR (41).

Immune function is negatively correlated with dysfunction of the HPA axis, which is prevalent in PTSD (11, 42, 43). In general, glucocorticoids limit inflammation by preventing the production and release of proinflammatory cytokines; however, in cases of excitotoxicity, increased glucocorticoid levels may cause the expression of proinflammatory cytokines (44-46). In addition, the neuroprogression associated with PTSD and other similar disorders has been linked to the inhibition of neurogenesis and promotion of neuronal mortality by microglial cytokines (47, 48).

Glucocorticoid resistance can be induced by pro-inflammatory cytokines such as IL-1 e IL-6 (49, 50).The heightened vulnerability to inflammation can be ascribed to dysfunctions within the hypothalamic-pituitary-adrenal (HPA) axis, occurring at various levels, including the hypothalamic corticotropin-releasing hormone (CRH), pituitary adrenocorticotropic hormone (ACTH), and adrenal glucocorticoid secretion. These dysfunctions can lead to a general deficiency in cortisol production or to a reduction in local factors that influence glucocorticoid availability (43). 

To examine the potential anti-inflammatory effect of tBHQ on the reduction of PTSD symptoms, pro-inflammatory and anti-inflammatory cytokines were assessed. In the two brain regions studied, rats with PTSD had higher levels of the pro-inflammatory cytokine IL-6. Elevated levels of IL-6 have been identified in individuals with PTSD (51, 52), which is consistent with the present findings. Moreover, numerous studies show that anti-inflammatory medications decrease IL-6 expression. Other research groups have also found that the anti-inflammatory cytokine IL-10 is diminished in PTSD (52). They concur with our findings that PTSD significantly reduces the expression of IL-10 in the amygdala and hippocampus. The increased expression of IL-10 following tBHQ treatment is intriguing, as previous research has demonstrated that anti-inflammatory medications reduce neuroinflammation.

It has been demonstrated that the transcription factor Nrf2, which is critical for defense against oxidative and xenobiotic stressors, can reduce inflammation. Nrf2 insufficiency leads to the development of autoimmune symptoms and inflammation. Furthermore, it is postulated that NF-κB signaling exerts a substantial influence on the synthesis of pro-inflammatory cytokines. Inducers of Nrf2 can modulate BDNF in order to stimulate the anti-inflammatory phenotype of microglia and inhibit the pro-inflammatory phenotype, according to recent research. 

In order to induce peripheral inflammation, Ghosh *et al*. injected LPS into the abdominal cavity of Swiss albino mice. This resulted in the development of depression-like symptoms, increased pro-inflammatory cytokine levels, and microglia activation, and activation of the NF-κB-p65 pathway. However, the administration of tBHQ inhibited alterations in the hippocampus’s autophagy and cell death pathways and reversed symptoms resembling depression. This was achieved by stimulating Nrf2-dependent gene expression. Furthermore, through the activation of the Nrf2/ARE pathway, tBHQ has the potential to mitigate depressive and anxious behaviors in mice (37).

tBHQ: Tert-butylhydroquinone

A. Effects of tBHQ on rat Anxiety Index in the EPM. B and C. Effects of tBHQ on B) immobility time and C) swimming time in the FST

The data were expressed as means ± SD (n = 10 rats per group). D. Effects of tBHQ on the concentration of corticosterone in serum. The data were expressed as means ± SD (n = 3 rats per group). **P< 0.01, ***P<0.001.

A) MAO-A in amygdala, B) MAO-B in amygdala, C) MAO-A in hippocampus, D) MAO-B in hippocampus. The data were expressed as means ± SD (n = 3 rats per group). *P<0.05, ***P<0.001. tBHQ: Tert-butylhydroquinone; MAOs: MAO-A and MAO-B

A) in amygdala and B) in hippocampus. The data were expressed as means ± SD (n = 3 rats per group), **P<0.01, ***P<0.001. tBHQ: Tert-butylhydroquinone; GR: glucocorticoid receptor

A) IL-6 in amygdala, B) IL-6 in hippocampus, C) IL-10 in amygdala, D) IL-10 in hippocampus. The data were expressed as means ± SD (n = 3 rats per group). **P<0.01, ***P<0.001.tBHQ: Tert-butylhydroquinone; IL-6: Interleukin 6, IL-10: Interleukin 10

## Conclusion

These antidepressant and anxiolytic effects were correlated with serum corticosterone levels, MAO concentrations, and GR expression in the amygdala and hippocampus. Furthermore, the findings revealed that tBHQ had a significant impact on the neuroinflammatory response, which was related to IL-6 suppression and IL-10 activation in both the amygdala and the hippocampus. These novel findings indicated that the anti-neuroinflammatory function of tBHQ ameliorates PTSD-induced depression and anxiety-like behaviors.

## Declaration

We have not used any AI tools or technologies to prepare this manuscript.
